# Nurses’ Perceptions of Authentic Leadership and Quality of Nursing Care: The Mediating Role of Psychological Empowerment

**DOI:** 10.1155/jonm/8865596

**Published:** 2025-12-18

**Authors:** Chiu-Shu Fang, Cheng-Hsien Li, Su-Chiu Hsiao, Chun-Chang Lin, Fang-Ming Hwang, Fan-Hao Chou, Shu-Ching Ma

**Affiliations:** ^1^ Department of Nursing, Shu-Zen Junior College of Medicine and Management, Kaohsiung, Taiwan, szmc.edu.tw; ^2^ Institute of Human Resource Management, National Sun Yat-sun University, Kaohsiung, Taiwan; ^3^ Department of Nursing, Chi-Mei Medical Center, Liouying, Tainan, Taiwan, chimei.org.tw; ^4^ Department of Nursing, Chang Jung Christian University, Tainan, Taiwan, cjcu.edu.tw; ^5^ Department of Nursing, Chi-Mei Medical Center, Chiali, Tainan, Taiwan, chimei.org.tw; ^6^ Department of Education, National Chiayi University, Chiayi, Taiwan, ncyu.edu.tw; ^7^ Center for Medical Education and Humanizing Health Professional Education, Kaohsiung Medical University, Kaohsiung, Taiwan, kmu.edu.tw; ^8^ College of Nursing, Kaohsiung Medical University, Kaohsiung, Taiwan, kmu.edu.tw; ^9^ Department of Nursing, Chi-Mei Medical Center, Yongkang, Tainan, Taiwan, chimei.org.tw; ^10^ Department of Senior Welfare and Services, Southern Taiwan University of Science and Technology, Tainan, Taiwan, stust.edu.tw

**Keywords:** authentic leadership, cross-sectional study, hospital nurses, psychological empowerment, quality of nursing care, structural equation modeling

## Abstract

**Background:**

Psychological empowerment plays a crucial role in influencing both the quality of nursing care and nurses’ self‐perceptions. However, limited research has examined its mediating effect on the relationship between nurses’ perceptions of authentic leadership and the quality of care provided. This study aimed to test whether psychological empowerment mediates the relationship between nurses’ perceptions of their supervisors’ authentic leadership and the perceived nursing care quality. The findings are intended to provide both practical strategies and theoretical insights for improving the quality of nursing care.

**Methods:**

This cross‐sectional study followed the STROBE reporting guidelines. An online survey was conducted between July and August 2022, yielding 944 complete responses (response rate: 65.42%) from nurses at three hospitals in Taiwan. Data were collected using a structured online questionnaire measuring authentic leadership, psychological empowerment, and perceived nursing care quality. Structural equation modeling (Mplus 8.10) was used to assess the mediating effect of psychological empowerment.

**Results:**

Significant positive relationships were found between nurses’ perceptions of authentic leadership and psychological empowerment, as well as between psychological empowerment and perceived nursing care quality. Psychological empowerment fully mediated the relationship between authentic leadership and perceived nursing care quality, indicating a significant indirect effect.

**Conclusions:**

Nurses’ perceptions of authentic leadership positively influenced their psychological empowerment, which, in turn, enhanced their evaluation of nursing care quality. Psychological empowerment thus serves as a key mediator in shaping nurses’ perceptions of nursing care quality.

**Implications for Nursing Management:**

Nursing managers should foster authentic leadership practices and implement empowerment‐oriented interventions to strengthen nurses’ motivation, autonomy, and engagement, thereby improving the overall quality of nursing care.

## 1. Introduction

In today’s increasingly complex healthcare systems, maintaining the quality of nursing care has become an urgent global priority. Nurses are frequently exposed to high psychological demands, heavy workloads, and ethical challenges that can adversely affect both their well‐being and patient outcomes [1]. Leadership is widely recognized as one of the most critical factors shaping nurses’ work environments and influencing care quality [2]. Among contemporary leadership models, authentic leadership—characterized by self‐awareness, transparency, balanced decision‐making, and moral integrity—has emerged as a key approach to promoting trust, engagement, and ethical practice within healthcare teams [3]. However, despite growing evidence linking authentic leadership to positive nursing and organizational outcomes, the underlying psychological processes through which it enhances nurses’ perceptions of care quality remain insufficiently understood.

Far less attention has been given to psychological empowerment—the intrinsic motivational process that explains how nurses translate leadership support into high‐quality practice [4]. To address this gap, the present study investigated psychological empowerment as a mediating mechanism linking authentic leadership to nurses’ perceived quality of nursing care. Integrating self‐determination theory and authentic leadership theory, this study contributes to the theoretical development of nursing leadership by clarifying how authentic leaders foster psychological empowerment and intrinsic motivation. The findings will also offer practical guidance for nurse managers seeking to implement empowerment‐driven leadership strategies that enhance nurse engagement, retention, and care quality.

## 2. Background

### 2.1. Direct Relationship: Authentic Leadership and Quality of Nursing Care

Authentic leadership has attracted increasing attention in nursing management because it emphasizes self‐awareness, relational transparency, moral perspective, and balanced decision‐making. Such leadership behaviors foster psychological safety, open communication, and an ethical work climate, which directly enhance nurses’ engagement, job satisfaction, and perceived quality of care [3]. Empirical evidence further suggests that nurses who perceive their leaders as authentic report fewer missed care incidents, demonstrate greater organizational commitment, and have greater trust in their institutions [5]. Similarly, Teng et al. [6] demonstrated that authentic leadership enhances nurses’ psychological well‐being and organizational performance by creating transparent and empowering work environments. Collectively, these findings indicate that authentic leadership is a key determinant of nursing care quality. By modeling ethical integrity and fostering mutual respect, authentic leaders inspire nurses to uphold professional standards and deliver high‐quality and patient‐centered care.

 H1. Nurses’ perceptions of authentic leadership are positively associated with their perceived quality of nursing care.

### 2.2. Authentic Leadership and Psychological Empowerment

Beyond its direct effects, authentic leadership may enhance nurses’ psychological empowerment—a motivational state characterized by meaning, competence, self‐determination, and impact [4]. Authentic leaders foster empowerment by promoting transparent communication, encouraging participation in decision‐making, and demonstrating genuine concern for staff development, thereby fulfilling nurses’ psychological needs for autonomy, competence, and relatedness [3, 7].

Recent empirical studies have further substantiated this association. Dirik and Seren Intepeler [8] found that authentic leadership training significantly increased nurses’ levels of psychological empowerment and improved their perceptions of the patient safety climate. Similarly, Sahraei Beiranvand et al. [9] demonstrated that authentic and ethical leadership enhanced nurses’ psychological empowerment by fostering transparency, shared decision‐making, and moral integrity. Taken together, these findings suggest that authentic leaders strengthen empowerment by cultivating trust, integrity, and participative decision‐making, thereby creating a work environment conducive to professional growth, autonomy, and intrinsic motivation.

 H2. Nurses’ perceptions of authentic leadership are positively associated with psychological empowerment.

### 2.3. Psychological Empowerment and Quality of Nursing Care

Psychological empowerment has been identified as a critical factor influencing nurses’ work engagement, professional performance, and perceptions of care quality. Empowered nurses exhibit greater autonomy, responsibility, and initiative in clinical decision‐making, which contribute to improved patient outcomes [10]. Empirical evidence supports this link. Huang et al. [11] reported that empowerment‐focused interventions significantly enhanced nurses’ professional efficacy and perceived quality of care. Zhuang et al. [12] further showed that psychological empowerment positively influences nurses’ thriving at work, which, in turn, is associated with higher quality of nursing services. Malak and Abu Safieh [13] found a direct positive association between work‐related psychological empowerment and nurses’ perceived quality of care in critical care settings. Similarly, Jin et al. [14] demonstrated that empowered nurses were more likely to remain in their profession, experience lower burnout, and provide better patient care. Collectively, these findings underscore psychological empowerment as a key determinant of nursing care quality and as a psychological mechanism through which nurses translate intrinsic motivation and autonomy into effective clinical practice.

 H3. Psychological empowerment is positively associated with nurses’ perceived quality of nursing care.

### 2.4. Psychological Empowerment as Mediator

Building upon the preceding relationships, psychological empowerment may serve as a mediating mechanism through which authentic leadership enhances nurses’ perceived quality of care. Authentic leaders foster empowerment by promoting transparency, fairness, and shared decision‐making, which in turn enhances nurses’ intrinsic motivation and engagement in providing high‐quality care [15]. Recent research has supported this indirect mechanism. Dirik and Seren Intepeler [8] demonstrated that authentic leadership training not only improved nurses’ perceptions of leadership authenticity but also elevated their psychological empowerment and safety climate, suggesting that empowerment acts as a pathway through which leadership influences outcomes. Similarly, Uluturk et al. [16] confirmed that psychological empowerment mediates the effect of authentic leadership on employees’ work outcomes, reinforcing its pivotal role in translating leader behaviors into performance improvement. Kim and Han [17] earlier found that nurse managers’ authentic leadership enhanced nursing performance through full mediation by psychological empowerment, providing foundational evidence for this mechanism. Collectively, these findings indicate that psychological empowerment functions as an essential psychological bridge linking authentic leadership to nursing care quality by increasing nurses’ sense of autonomy, competence, and professional engagement.

 H4. Psychological empowerment mediates the relationship between nurses’ perceptions of authentic leadership and their perceived quality of nursing care.

**Figure 1:**
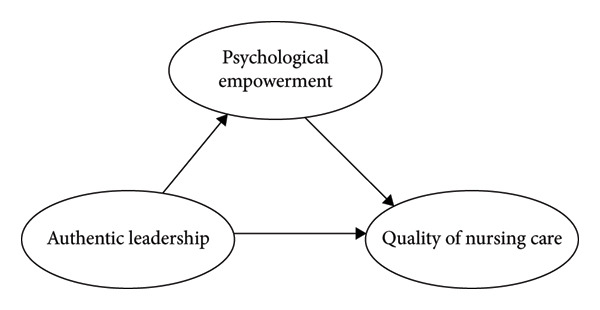
Hypothesized model.

Although prior research has examined the associations among authentic leadership, empowerment, and various organizational outcomes, limited empirical evidence has specifically explored the psychological mechanism through which authentic leadership influences nurses’ perceptions of nursing care quality. Existing studies have primarily focused on structural empowerment, work engagement, or retention, leaving a critical gap in understanding how psychological empowerment functions as an internal motivational process linking leadership behaviors to nursing care quality outcomes. In this study, we aimed to fill this gap by empirically testing a mediated model that positions psychological empowerment as a central pathway between authentic leadership and the perceived quality of nursing care. Integrating self‐determination theory and authentic leadership theory, this research advances the theoretical understanding of how authentic leaders cultivate psychological conditions that enhance nurses’ motivation, autonomy, and professional performance. From a practical standpoint, the findings provide nursing managers with evidence‐based insights into how to foster empowerment‐driven leadership practices that strengthen nurse engagement and elevate the overall quality of patient care. The conceptual model of this relationship is illustrated in Figure 1.

## 3. Materials and Methods

### 3.1. Study Design

This study employed a cross‐sectional design and adhered to the STROBE guidelines for reporting observational studies. The study aimed to evaluate the relationship between nurses’ perceived authentic leadership and their self‐assessed quality of nursing care, as well as to explore the mediating role of psychological empowerment in this relationship.

### 3.2. Setting and Participants

Data were collected from July to August 2022 across three hospitals in southern Taiwan, including a medical center, a regional hospital, and a district hospital. An online structured questionnaire survey was used for data collection. The inclusion criteria required participants to be (1) aged 20 years or older and (2) have at least 6 months of clinical work experience. Nurses were excluded if they (1) were on long‐term leave; (2) held a position of deputy head nurse or higher; or (3) did not directly provide care to hospitalized patients. A total of 1443 eligible nurses were invited to participate; 951 questionnaires were returned. Of these, 944 were fully completed and included in the final analysis, resulting in an effective response rate of 65.42%. Seven questionnaires were excluded due to missing data.

Since G∗Power does not directly estimate the sample size for structural equation modeling (SEM) [18], this study determined the required sample size based on several practical recommendations. Nevitt and Hancock [19] suggested that an optimal SEM sample size ranges from 200 to 1000 participants. Kline [20] further suggested that the minimum required sample size should be determined using an *N*:*q* ratio of at least 10:1, where *N* represents the number of cases and *q* denotes parameters to be estimated. Given that this study includes 72 model parameters, a minimum of 720 participants was deemed necessary.

### 3.3. Data Collection

The measurement tools used in this study included the Authentic Leadership Questionnaire (ALQ), Psychological Empowerment Scale (PES), and Quality of Nursing Care Scale (QNC Scale). The ALQ and PES scales were obtained with permission for both Chinese and English versions, while the QNC Scale was translated and back‐translated following Brislin’s [21] model after receiving authorization, ensuring semantic equivalence and cultural appropriateness for the Chinese version. Following approval from the Institutional Review Board (IRB) and consent from the nursing departments of the three participating hospitals, an online questionnaire was distributed via QR code to the target nursing units. Nurses accessed the survey by scanning the QR code and completed the questionnaire, which took approximately 15 min. A total of 1443 survey links were distributed to achieve an adequate sample size for subsequent analysis.

### 3.4. Measures

#### 3.4.1. Sociodemographic Characteristics

The sociodemographic questionnaire was used to collect data on participants’ age, gender, marital status, educational level, hospital type, years of clinical experience, professional advancement level, and work shift pattern. These variables were included as control variables to account for their potential influence on the relationships among the study variables, particularly the quality of nursing care [22–26].

#### 3.4.2. ALQ

This study utilized the 16‐item ALQ developed by Walumbwa et al. [27] to assess nurses’ perceptions of their supervisors’ authentic leadership. The questionnaire consisted of four subdimensions: self‐awareness, relational transparency, balanced processing, and internalized moral perspective. Responses were rated on a five‐point Likert scale, ranging from 0 (*strongly disagree*) to 4 (*strongly agree*), with higher scores indicating a stronger perception of authentic leadership. The reliability of the Chinese version of the ALQ, as measured by Cronbach’s α, was reported to be 0.85 [28].

#### 3.4.3. PES

This study employed the 12‐item PES developed by Spreitzer [29] to assess nurses’ psychological empowerment. The scale comprises four dimensions: meaning, competence, self‐determination, and impact. Responses were rated on a seven‐point Likert scale ranging from 1 (*strongly disagree*) to 7 (*strongly agree*), with higher scores indicating greater psychological empowerment. The reliability of the total scale, as measured by Cronbach’s α, was reported to be 0.91 among U.S. nurses [30], while the Chinese version demonstrated a Cronbach’s α of 0.85 in a nursing sample [31].

#### 3.4.4. QNC Scale

This study adopted the 25‐item scale of perception of nursing activities that contribute to nursing care quality (EPAECQC Scale), developed by Martins et al. [32], to assess nurses’ perceptions of the quality of nursing care they provide. The scale consisted of seven subdimensions: patient satisfaction, health promotion, prevention of complications, well‐being and self‐care, functional readaptation, care organization, and responsibility and rigor. Responses were rated on a four‐point Likert scale ranging from 1 (*never*) to 4 (*always*), with higher scores indicating a greater perceived quality of nursing care. The original version of the scale demonstrated high reliability, with a Cronbach’s α of 0.94 [32].

### 3.5. Data Analysis

Descriptive statistics were conducted using SPSS Version 25.0 (SPSS Inc., Chicago, IL, USA) to assess the means, standard deviations (SDs), skewness, and kurtosis of each observed variable. Following the recommendations of Kwak and Park [33], the data were considered approximately normally distributed if skewness values were within ±2 and kurtosis values within ±7. In this study, the absolute values of skewness ranged from 0.001 to 1.690, while the absolute values of kurtosis ranged from 0.005 to 3.373, indicating that the observed variables were approximately normally distributed. Harman’s single‐factor test was employed to examine common method variance (CMV). If the total variance explained by a single factor exceeded 50%, CMV was considered a potential concern [34]. In the present study, the cumulative variance explained by a single factor was 34.62%, suggesting that CMV was not a significant concern.

SEM was conducted using Mplus Version 8.10 [35] to evaluate the overall model structure and assess the mediating effects. Given the approximately normal distribution of all observed variables, the maximum likelihood (ML) estimation method was applied to estimate the model parameters [35]. This study followed the two‐step approach recommended by Anderson and Gerbing [36] for SEM validation. First, the measurement model was validated using confirmatory factor analysis (CFA) to verify the reliability and validity of the latent constructs. Second, the structural model was tested to examine the hypothesized relationships among the latent variables [20]. To enhance the robustness of parameter estimation and account for the potential nonnormality of the structural path coefficients, particularly indirect effects, a bias‐corrected bootstrap procedure with 1000 resamples was employed [37]. This approach was used to generate 95% confidence intervals (CIs) for all structural path coefficients, including both direct and indirect effects, to assess their statistical significance.

The model fit was evaluated using multiple indices, including the chi‐square to degrees of freedom ratio (*χ* ^2^/df), comparative fit index (CFI), Tucker–Lewis Index (TLI), RMSEA, and standardized root mean square residual (SRMR). According to Hair et al.’s recommendations [38], a *χ* ^2^/df value less than three indicated a good model fit. CFI and TLI values greater than 0.90, along with RMSEA and SRMR values less than 0.08, indicated an acceptable model fit. Furthermore, the R‐squared (*R* ^2^) value was used to assess the proportion of variance explained by the model for each endogenous construct, with higher *R* ^2^ values indicating greater explanatory power [39]. All statistical analyses were conducted using two‐tailed tests; statistical significance was set at *p* < 0.05.

### 3.6. Ethical Considerations

This study adhered to the ethical principles of the Declaration of Helsinki and received approval from the IRB of a medical center on June 9, 2022 (IRB no. 11104–014), with additional approvals obtained from two other hospitals within the same healthcare system. Participants accessed the online questionnaire via a QR code provided by the research team. Before beginning the survey, they were required to read an electronic information sheet describing the study’s purpose, procedures, minimal risks, confidentiality protections, and the voluntary nature of participation.

Written informed consent was obtained electronically. Participants acknowledged their consent by clicking the “Next” button after reviewing the online information sheet. Individuals who did not agree to participate could simply close the webpage without providing any data. Data were collected anonymously through the SurveyCake platform, which ensured encrypted data transmission, confidentiality, and restricted access to authorized researchers only. As a token of appreciation for their participation, respondents who completed the questionnaire received a convenience store gift card worth NT$100 (approximately US$3.30).

## 4. Results

### 4.1. Sociodemographic Characteristics of Participants

The sociodemographic characteristics of the participants are presented in Table 1. A total of 944 nurses were included in this study. More than half (54.8%) worked in a medical center, and 93% were female participants. Additionally, 65.7% were single. The mean age of the participants was 31.91 years (SD = 7.00), and 52.9% aged 30 years or younger. The average length of clinical experience was 7.35 years (SD = 6.06), and 97.8% held a bachelor’s degree or higher. Regarding work settings, 62.5% of nurses were employed in general wards, followed by 37.5% in emergency departments or intensive care units, and 2.9% in psychiatric wards. More than half (53.8%) were at clinical advancement level N2 or below, and 79% worked fixed rotating shifts.

**Table 1:** Sociodemographic characteristics of participants (*n* = 944).

Variables	*n*	%
Hospital level		
Medical center	517	54.8
Regional	328	34.7
District	99	10.5
Gender		
Male	66	7.0
Female	878	93.0
Age		
20–30	499	52.9
31–40	302	32.0
41–50	135	14.3
50–62	8	0.8
Mean ± SD	31.91 ± 7.00	
Working years		
Mean ± SD	7.35 ± 6.06	
Marital status		
Single	620	65.7
Married	309	32.7
Divorced or loss of a spouse		1.6
Education		
Associate degree	21	2.2
Bachelor degree	912	96.6
Master’s degree or above	11	1.2
Work unit		
General ward	590	62.5
ER or ICU	354	37.5
Professional advancement level		
N1	226	23.9
N2	282	29.9
N3	270	28.6
N4	166	17.6
Type of work		
Fixed day shift	278	29.4
Fixed evening shift	244	25.8
Fixed night shift	224	23.7
Rotating shifts	198	21.0

*Note*: SD, standard deviation; ER, emergency room; ICU, intensive care unit.

### 4.2. Measurement Model

The measurement model comprised three latent variables: authentic leadership (16 items), psychological empowerment (12 items), and quality of nursing care (25 items). First‐order CFA results indicated that all observed variables had statistically significant standardized factor loadings, ranging from 0.59 to 0.93. The model fit indices demonstrated an acceptable fit (*χ* ^2^/df = 3.008, CFI = 0.942, TLI = 0.935, SRMR = 0.035, and RMSEA = 0.046 [90% CI = 0.044, 0.048]).

Table 2 presents the correlation matrix of the latent variables, showing that all variables were significantly correlated, thereby supporting the establishment of structural paths among the latent variables. Convergent validity was supported, as the composite reliability (CR) values exceeded 0.70, the average variance extracted (AVE) values were above 0.50, and all standardized factor loadings (*λ*) were greater than 0.50 [40]. Furthermore, the square roots of the AVEs for each latent variable were greater than their correlations with any other latent variables, supporting discriminant validity [41].

**Table 2:** Discriminant validity for latent variables.

Latent variables	CR	AVE	1.	2.	3.
1. Authentic leadership	0.90	0.69	**(0.83)**		
2. Psychological empowerment	0.90	0.74	0.43^∗∗∗^	**(0.86)**	
3. Quality of nursing care	0.84	0.62	0.24^∗∗∗^	0.59^∗∗∗^	**(0.79)**

*Note:* The diagonal values in bold represent the square root of the AVE for each latent variable; CR, composite reliability; AVE, average variance extracted.

^∗^ *p* < 0.05.

^∗∗^ *p* < 0.01.

^∗∗∗^ *p* < 0.001.

### 4.3. Structural Model

**Figure 2:**
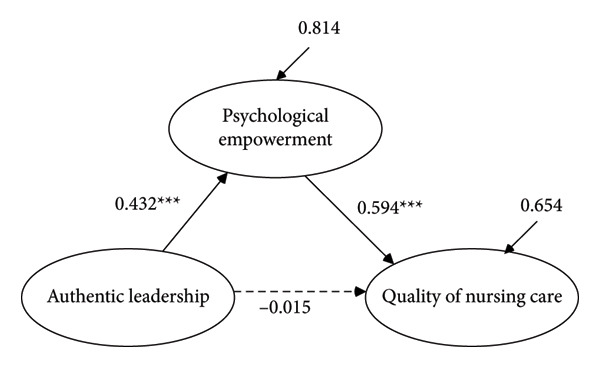
Standardized regression weights for the simplified structural model. *Note:* Ovals represent latent variables; solid lines indicate significant effects; and dashed lines indicate nonsignificant effects. ^∗^ *p* < 0.01, ^∗∗^ *p* < 0.05, and ^∗∗∗^ *p* < 0.001.

The hypothesized structural model, representing the relationships among latent variables, demonstrated a good fit with the observed data (*χ* ^2^/df = 3.416, CFI = 0.926, TLI = 0.921, RMSEA = 0.051 [90% CI: 0.049, 0.052], and SRMR = 0.050). The standardized structural path coefficients are illustrated in Figure 2. The direct effect of authentic leadership on the quality of nursing care was not statistically significant (*β* = −0.015, 95% CI [‐0.089, 0.058]); thus, Hypothesis 1 was not supported. In contrast, authentic leadership had a significant positive effect on psychological empowerment (*β* = 0.432, 95% CI [0.357, 0.499]), and psychological empowerment had a significant positive effect on the quality of nursing care (*β* = 0.594, 95% CI [0.527, 0.664]), supporting Hypotheses 2 and 3.

To verify the robustness of the model, an adjusted model was tested by controlling for all sociodemographic characteristics of the participants. The strength and direction of the relationships among the study variables remained consistent with the unadjusted model. Furthermore, the model fit indices were satisfactory (*χ* ^2^/df = 2.843, *p* < 0.001, CFI = 0.913, TLI = 0.909, RMSEA = 0.044 [90% CI: 0.043–0.046], and SRMR = 0.056). Among the control variables, hospital type (regional hospitals; *β* = −0.103, *p* = 0.034), gender (female; *β* = 0.073, *p* = 0.012), and work unit (wards; *β* = 0.072 and *p* = 0.015) showed significant associations with nursing care quality. The adjusted model explained 37.5% of the variance in the quality of nursing care (*R* ^2^ = 0.375). For the sake of parsimony, the mediation analysis was primarily reported based on the unadjusted model, which did not include control variables.

The indirect effect of authentic leadership on nursing care quality through psychological empowerment was statistically significant (*β* = 0.256, 95% CI [0.206, 0.312]), supporting Hypothesis 4 and confirming the mediating role of psychological empowerment. Furthermore, the direct effect of authentic leadership on nursing care quality remained nonsignificant after accounting for the mediator, indicating full mediation. These findings suggest that although authentic leadership does not directly influence perceived nursing care quality, its effect is fully mediated by psychological empowerment. The model explained 34.6% of the variance in the quality of nursing care (*R* ^2^ = 0.346). These results are summarized in Table 3.

**Table 3:** Standardized direct and indirect effects with 95% confidence intervals for the hypothesized paths in the structural model (*n* = 944).

Model pathways	Standardized *β*	Stand error	*p* value	95% CI	Finding
Direct effect					
ALQ⟶QNC	−0.015	0.037	0.685	[−0.089, 0.058]	Not support
Indirect effect					
ALQ⟶PE⟶QNC	0.256^∗∗∗^	0.026	< 0.001	[0.206, 0.312]	Support
Total indirect effect	0.256^∗∗∗^	0.026	< 0.001	[0.206, 0.312]	Support
Total effect	0.242^∗∗∗^	0.037	< 0.001	[0.168, 0.312]	Support

*Note*: Estimates are standardized coefficients. Confidence intervals were obtained using a 95% bias‐corrected bootstrap with 1000 resamples. ALQ, authentic leadership; PE, psychological empowerment; QNC, quality of nursing care; CI, confidence interval.

^∗^ *p* < 0.05.

^∗∗^ *p* < 0.01.

^∗∗∗^ *p* < 0.001.

## 5. Discussion

This study examined the relationships among nurses’ perceptions of authentic leadership, psychological empowerment, and nursing care quality, confirming the full mediating role of psychological empowerment in this relationship. Consistent with self‐determination theory [42] and authentic leadership theory [43], the findings highlight that authentic leadership enhances nurses’ perceptions of nursing care quality not directly but through intrinsic motivational processes that strengthen their sense of empowerment, competence, and autonomy. The majority of participants were female nurses (93%), which reflects the actual gender distribution of the nursing workforce in Taiwan, where more than 95.3% of registered nurses are women [44]. Therefore, this imbalance was expected and representative of the national nursing population.

Authentic leadership indirectly enhanced nurses’ perceived quality of nursing care through psychological empowerment. When nurses perceived higher levels of authentic leadership, they reported stronger feelings of meaning, competence, and self‐determination—core components of empowerment—which, in turn, strengthened their sense of providing high‐quality nursing care. These results highlight the psychological mechanism by which leadership behaviors influence care outcomes, extending prior research that has primarily focused on structural or contextual factors. These findings provide theoretical and managerial implications. To improve nursing care quality, healthcare organizations should foster both authentic leadership behaviors and empowerment‐driven environments that enable nurses to act with autonomy and purpose.

### 5.1. Authentic Leadership Does Not Directly Influence Nurse‐Perceived Quality of Nursing Care

The nonsignificant direct effect of authentic leadership on perceived care quality contrasts with findings in some earlier studies [3, 45], which reported positive direct associations between leadership and care quality or safety outcomes. However, the present findings align with those in research suggesting that leadership effects are often indirect and psychologically mediated rather than linear or immediate. For instance, Wong and Laschinger [46] and Fallatah and Laschinger [47] demonstrated that authentic leadership influenced job performance and satisfaction primarily through mediators such as empowerment, trust, and supportive work environments, indicating that leadership may shape care outcomes by transforming the psychological climate in which nurses operate rather than through direct behavioral control.

A plausible explanation for this pattern lies in both conceptual and methodological distinctions between studies. Prior research frequently examined leadership as an external managerial behavior and measured nursing care quality using single‐item or limited indicators, which might have overemphasized visible managerial effects. In contrast, the current study adopted a more comprehensive and multidimensional assessment using the 25‐item QNC Scale [32]. This scale captures complex aspects of care—such as patient satisfaction, health promotion, prevention of complications, well‐being and self‐care, functional recovery, organizational rigor, and professional responsibility—many of which are indirectly influenced by the internal psychological states of nurses. As Hair and Alamer [38] noted, multi‐item constructs provide greater reliability and construct validity, allowing for a more accurate representation of the subtle pathways linking leadership to care outcomes. Consequently, the comprehensive measurement approach used here might have revealed that authentic leadership exerts its influence primarily through psychological empowerment and motivation, rather than through direct managerial oversight of care delivery.

### 5.2. Authentic Leadership Significantly Enhances Psychological Empowerment

Consistent with previous findings [8, 9, 48, 49], authentic leadership was found to significantly enhance nurses’ psychological empowerment. Authentic leaders demonstrate transparency, moral integrity, and balanced decision‐making, thereby fostering a work climate of trust and participation [50].

According to self‐determination theory, these behaviors fulfill nurses’ basic psychological needs for autonomy, competence, and relatedness, which promote intrinsic motivation and empowerment [16, 51]. When nurses feel that their leaders acknowledge their contributions, involve them in meaningful decisions, and respect their professional autonomy, they experience greater empowerment and motivation to perform at a higher level. Empirical studies have also shown that authentic leadership increases nurses’ psychological empowerment, which in turn enhances their engagement, self‐efficacy, and overall job satisfaction [4, 8].

Taken together, these findings underscore that authentic leadership not only models ethical integrity but also strengthens nurses’ internal motivation by cultivating a psychologically empowering climate. Nurse managers who practice authentic leadership can, therefore, play a crucial role in creating environments that sustain empowerment and promote optimal care performance.

### 5.3. Psychological Empowerment Enhances Nurses’ Perceived Quality of Nursing Care

This study confirmed that psychological empowerment significantly predicts nurses’ perceived quality of nursing care, consistent with prior research emphasizing empowerment as a critical driver of care performance [10–13]. Empowered nurses exhibit higher levels of autonomy, accountability, and initiative, which directly translate into improved patient safety and satisfaction outcomes.

From a theoretical perspective, empowerment embodies both structural and psychological dimensions [52]. Although structural empowerment pertains to access to resources and information, psychological empowerment refers to the internalized sense of control and competence. These two dimensions are mutually reinforcing: structural supports foster psychological empowerment, which then enhances engagement and care delivery. Studies such as de Valle et al. [53] and Aydoğdu and Balsanelli [54] demonstrate that empowered nurses are more proactive in clinical decision‐making and more committed to continuous improvement. Therefore, cultivating psychological empowerment can serve as a bridge linking individual motivation with organizational performance.

### 5.4. Psychological Empowerment Fully Mediates the Relationship Between Authentic Leadership and Nurses’ Perceived Quality of Nursing Care

The full mediation effect observed in this study underscores that psychological empowerment is the core mechanism through which authentic leadership influences perceived nursing care quality. This finding aligns with recent evidence demonstrating that empowerment mediates the relationship between leadership styles and performance outcomes in nursing contexts [12, 55]. Dirik and Seren Intepeler [8] specifically demonstrated that authentic leadership training programs that enhance psychological empowerment led to improved patient safety outcomes, reinforcing the critical pathway identified in our study.

Beyond nursing contexts, further empirical evidence supports this mechanism beyond the nursing field. Almasradi et al. [56] found that authentic leadership did not directly influence socially responsible behavior but exerted its effect through full mediation by psychological empowerment and psychological capital, highlighting empowerment as a key psychological resource in leadership effectiveness. Consistent with these findings, Yu et al. [57] also confirmed the mediating role of psychological empowerment, emphasizing that nurse managers should recognize the importance of work engagement and continually enhance nurses’ sense of dignity and empowerment to improve nursing care quality.

Taken together, these results provide compelling evidence that authentic leadership influences nursing care quality primarily through its ability to foster nurses’ psychological empowerment—enhancing their sense of meaning, competence, self‐determination, and impact within their professional roles.

## 6. Implications for Nursing Management

The findings of this study provide both theoretical and practical insights into nursing management. Theoretically, the full mediation of psychological empowerment confirms its central role in explaining how authentic leadership influences nurses’ perceived quality of care. This finding supports self‐determination theory, which posits that autonomy‐supportive environments enhance intrinsic motivation and performance [58], and authentic leadership theory, which emphasizes that leaders’ self‐awareness, moral perspective, and transparency foster trust and empowerment [16]. Together, these frameworks clarify the motivational process through which authentic leadership enhances psychological empowerment and, consequently, nursing care quality.

Practically, the results suggest that authentic leadership alone is insufficient to improve nursing care quality unless accompanied by empowerment‐focused management strategies. Nurse managers should promote transparency, participative decision‐making, and recognition of nurses’ professional contributions to strengthen empowerment and engagement [8, 9]. Leadership development programs that cultivate self‐awareness and relational authenticity have been shown to enhance empowerment and psychological safety [59]. At the organizational level, providing nurses with opportunities for professional development and participatory decision‐making can foster empowerment, reduce burnout, and ultimately improve nursing care quality [14, 55]. Finally, given the predominance of female nurses in most countries, including Taiwan, empowerment‐oriented leadership may help address gendered expectations and hierarchical constraints within the nursing profession by fostering inclusion, respect, and shared authority [60]. Collectively, these insights underscore the importance of cultivating authentic, empowerment‐driven leadership to sustain a motivated and high‐performing nursing workforce.

## 7. Limitations

Although this study provides robust empirical support through SEM, several limitations should be acknowledged. First, the use of an online self‐reported questionnaire might have introduced response bias. The large number of items could have increased participants’ cognitive load and response fatigue; although a response‐time filter was applied to improve data quality, inattentive or careless responses cannot be entirely ruled out. Second, the extensive number of items increased model complexity, which may have affected the robustness of the SEM results. Third, due to the large sample size requirements of SEM, a nonrandom sampling method was adopted, recruiting nurses from three hospitals within the same healthcare system. This sampling approach may limit the generalizability of the findings to other regions or healthcare contexts. Finally, although the sample was predominantly female—reflecting the actual composition of Taiwan’s nursing workforce—the relatively small proportion of male nurses might limit the ability to fully capture potential gender differences in leadership perceptions and nursing care quality evaluations.

## 8. Conclusions

This study demonstrated that psychological empowerment fully mediated the relationship between nurses’ perceptions of authentic leadership and their perceived quality of nursing care. Authentic leadership did not exert a direct effect on nursing care quality; rather, it enhanced nurses’ psychological empowerment, which in turn indirectly improved their perceptions of nursing care quality. These findings highlight psychological empowerment as a key mechanism through which leadership behaviors translate into improved nursing care quality and provide valuable guidance for leadership development and nursing management practices.

## Disclosure

All authors agree to be accountable for all aspects of the work and have approved the final version for publication.

## Conflicts of Interest

The authors declare no conflicts of interest.

## Author Contributions

Chiu‐Shu Fang: conceptualization, data curation, formal analysis, methodology, writing–original draft preparation, and writing–review and editing.

Cheng‐Hsien Li: data curation, formal analysis, visualization, writing–original draft preparation, and writing–review and editing.

Su‐Chiu Hsiao: investigation, resource management, and writing–review and editing.

Chun‐Chang Lin: investigation, resource management, and writing–review and editing.

Fang‐Ming Hwang: data curation, formal analysis, validation, and writing–review and editing.

Fan‐Hao Chou: conceptualization, methodology, project administration, supervision, writing–original draft preparation, and writing–review and editing.

Shu‐Ching Ma: investigation, resource management, writing–original draft preparation, and writing–review and editing.

All authors critically revised the manuscript for important intellectual content. Dr. Fan‐Hao Chou and Dr. Shu‐Ching Ma share equal responsibility as corresponding authors.

## Funding

This research did not receive any specific grant from funding agencies in the public, commercial, or not‐for‐profit sectors.

## Data Availability

The data supporting the findings of this study are available from the corresponding author upon reasonable request.
